# Osteopontin and Other Regulators of Angiogenesis and Fibrogenesis in the Vitreous from Patients with Proliferative Vitreoretinal Disorders

**DOI:** 10.1155/2012/493043

**Published:** 2012-09-29

**Authors:** Ahmed M. Abu El-Asrar, Mohd Imtiaz Nawaz, Dustan Kangave, Mohammed Mairaj Siddiquei, Karel Geboes

**Affiliations:** ^1^Department of Ophthalmology, College of Medicine, King Saud University, Riyadh, Saudi Arabia; ^2^Department of Ophthalmology, King Abdulaziz University Hospital, Old Airport Road, P.O. Box 245, Riyadh 11411, Saudi Arabia; ^3^Laboratory of Histochemistry and Cytochemistry, University of Leuven, Leuven, Belgium

## Abstract

The aim of this study was to determine the levels of the angiogenic and fibrogenic factors osteopontin (OPN), high-mobility group box-1 (HMGB1), and connective tissue growth factor (CTGF) and the antiangiogenic and antifibrogenic pigment epithelium-derived factor (PEDF) in the vitreous fluid from patients with proliferative diabetic retinopathy (PDR), proliferative vitreoretinopathy (PVR), and rhegmatogenous retinal detachment with no PVR (RD). Vitreous samples from 48 PDR, 17 PVR and 30 RD patients were studied by enzyme-linked immunosorbent assay. OPN, HMGB1, CTGF, and PEDF levels were significantly higher in PDR patients than in RD patients (*P* < 0.001; 0.002; <0.001; <0.001, resp.). CTGF and PEDF levels were significantly higher in PVR patients than in RD patients (*P* < 0.001; 0.004, resp.). Exploratory logistic regression analysis identified significant associations between PDR and high levels of HMGB1, CTGF and PEDF, between PDR with active neovascularization and high levels of CTGF and PEDF, and between PDR with traction retinal detachment and high levels of HMGB1. In patients with PDR, there were significant correlations between the levels of PEDF and the levels of OPN (*r* = 0.544, *P* = 0.001), HMGB1 (*r* = 0.719, *P* < 0.001), and CTGF (*r* = 0.715, *P* < 0.001). In patients with PVR, there were significant correlations between the levels of OPN and the levels of HMGB1 (*r* = 0.484, *P* = 0.049) and PEDF (*r* = 0.559, *P* = 0.02). Our findings suggest that OPN, HMGB1, and CTGF contribute to the pathogenesis of proliferative vitreoretinal disorders and that increased levels of PEDF may be a response to counterbalance the activity of angiogenic and fibrogenic factors in PDR and PVR.

## 1. Introduction

 Ischemia-induced pathologic growth of new blood vessels and expansion of extracellular matrix (ECM) in association with the outgrowth of fibrovascular epiretinal membranes at the vitreoretinal interface is the pathological hallmark in proliferative diabetic retinopathy (PDR) and often leads to catastrophic loss of vision due to vitreous hemorrhage and/or traction retinal detachment. Proliferative vitreoretinopathy (PVR) is a process of fibrocellular proliferation on either sides of the retina that may complicate rhegmatogenous retinal detachment. The formation and gradual contraction of epiretinal membranes causes a marked distortion of the retinal architecture and results in complex retinal detachments that are difficult to repair.

 Angiogenesis, the growth of new vascular networks from preexisting ones, is under tight regulation by a dynamic balance between angiogenic stimulators and inhibitors [1]. The biological process of fibrosis, typically associated with an abnormal accumulation of ECM, occurs in response to various stimuli in many biological systems. The key cellular mediator of fibrosis is the myofibroblast, a cell type differentiated from quiescent fibroblasts. These are contractile cells, characterized by the expression of *α*-smooth muscle actin (*α*-SMA), and their presence is a marker of progressive disease. They have the capacity to produce several ECM components including collagen resulting in fibrosis [2]. Previous studies have shown that *α*-SMA-expressing myofibroblasts are the principal cellular component of PDR and PVR epiretinal membranes [3–6]. Inflammation, angiogenesis, and fibrosis are processes involved in the pathogenesis of proliferative vitreoretinal disorders, and the interplay between these events is under intense investigation [3–8]. A number of proinflammatory, proangiogenic, profibrogenic, and immunomodulating factors may be linked to the development and progression of proliferative vitreoretinal disorders, such as osteopontin (OPN), high-mobility group box-1 (HMGB1), connective tissue growth factor (CTGF), and pigment epithelium-derived factor (PEDF).

 Osteopontin is a phosphorylated acidic arginine-glycine-aspartate- (RGD-)containing glycoprotein that exists both as an immobilized ECM component and as a soluble, multifunctional, proinflammatory cytokine that plays important roles in promoting inflammation [9, 10], tissue remodeling, fibrosis [9, 11–14], and angiogenesis [15–18]. Many of these effects are mediated by the binding of OPN to CD44 receptors and the surface integrin receptor *α*_*v*_ *β*_3_ [15, 16, 19]. HMGB1 is a nonhistone DNA-binding nuclear protein that is highly conserved during evolution. Necrotic cell death can result in passive leakage of HMGB1 from the cell as the protein is then no longer bound to DNA. In addition, HMGB1 can be actively secreted by different cell types, including activated monocytes and macrophages, mature dendritic cells, natural killer cells, and endothelial cells. Extracellular HMGB1 functions as a proinflammatory cytokine [20–23] and exhibits angiogenic [24–27] and fibrogenic [28–31] effects. CTGF is a cysteine-rich secretory protein that functions as a downstream mediator of transforming growth factor-*β* action on connective tissue cells [32]. It acts as a fibroblast chemoattractant and mitogen and also stimulates the production of ECM components in various fibroblast cultures [32–34]. In addition, *in vitro* and *in vivo* studies demonstrated that CTGF exhibits angiogenic activities [35, 36]. 

 PEDF is a 50 KDa secreted glycoprotein that belongs to the noninhibitory serpin family group. PEDF has been described as a natural inhibitor of both physiological and pathological angiogeneses with antioxidant, and anti-inflammatory effects. It has been demonstrated to function as a potent and broadly acting neurotrophic and neuroprotective factor that induces cell differentiation and protects neurons in the brain, eye, and spinal cord against a wide range of neurodegenerative insults [37, 38]. In addition, PEDF was recently shown to have antifibrogenic activity [39]. 

 To address mechanisms involved in the pathogenesis of proliferative vitreoretinal disorders and to identify molecular targets for treatment and/or preventive intervention, we measured the levels of OPN, HMGB1, CTGF, and PEDF in the vitreous fluid from patients with PDR, PVR, and rhegmatogenous retinal detachment with no PVR (RD). In addition, we correlated their levels with PDR clinical disease activity.

## 2. Materials and Methods

### 2.1. Vitreous Samples

Undiluted vitreous fluid samples (0.3–0.6 mL) were obtained from 48 patients with PDR, 17 patients with PVR, and 30 patients with RD during pars plana vitrectomy. The indications for vitrectomy in patients with PDR were traction retinal detachment and/or nonclearing vitreous hemorrhage. In patients with PDR, the severity of retinal neovascular activity was graded clinically at the time of vitrectomy using previously published criteria [40]. Neovascularization was considered active if there were visible perfused new vessels on the retina or optic disc present within tractional epiretinal membranes. Neovascularization was considered inactive (involuted) if only nonvascularized, white fibrotic epiretinal membranes were present. Active PDR was present in 28 patients, and inactive PDR was present in 20 patients. Traction retinal detachment was present in 21 patients. Vitreous samples were collected undiluted by manual suction into a syringe through the aspiration line of vitrectomy, before opening the infusion line. The samples were centrifuged (500 rpm for 10 min, 4°C), and the supernatants were aliquoted and frozen at −80°C until assay. The study was conducted according to the tenets of the Declaration of Helsinki, and informed consent was obtained from all patients. The study was approved by the Research Centre, College of Medicine, King Saud University.

### 2.2. Enzyme-Linked Immunosorbent Assay Kits

Enzyme-linked immunosorbent assay (ELISA) kit for human OPN (Human Osteopontin, DuoSet, Cat no. DY1433) was purchased from R&D Systems, Minneapolis, MN. An ELISA kit for HMGB1 (human high-mobility group box-1, Cat no. ST51011) was purchased from IBL International GMBH (Hamburg, Germany). ELISA kits for human CTGF (human connective tissue growth factor, Cat No: E0010h) and human PEDF (human pigment epithelium-derived factor, Cat no. CSB-E08818h) were purchased from USCN life science & Tech Co., Ltd. and Cusabio Biotech Co., Ltd. Wuhan, China, respectively. The minimum detection limit of each ELISA kit for OPN, HMGB-1, CTGF, and PEDF are 2.5, 200, 15.6, and 40 picograms/mL (pg/mL), respectively. The ELISA plate readings were done using FLUOstar Omega-Miroplate reader from BMG Labtech, Offenburg, Germany. 

### 2.3. Measurement of Human OPN, HMGB-1, CTGF, and PEDF

The quantification of human OPN, HMGB-1, CTGF and PEDF in the vitreous fluid was determined using ELISA kits according to the manufacturer's instruction. For each ELISA kit, the undiluted standard serves as the highest standard and calibrator diluents serve as the blank. Depending upon the detection range for each ELISA kit and the expression level of the particular molecule, vitreous samples were either directly used or diluted with calibrator diluents supplied with ELISA kit. 

For the measurement of OPN, 100 *μ*L of 1000-fold diluted vitreous samples were added into each of the ELISA plate for the analysis. For the quantification of HMGB1, 60 *μ*L of diluent buffer was added to each well of microtiter plate followed by the addition of 40 *μ*L of standard, positive control, and vitreous fluid. For the measurement of CTGF, and PEDF, 100 *μ*L of 3-fold and 6-fold diluted vitreous were used, respectively, for ELISA assay. 

As instructed in the kit manual, vitreous samples were incubated into each well of ELISA plates. Antibodies against OPN, HMGB1, CTGF and PEDF conjugated to horseradish peroxidase were added to each well of the ELISA plate. After incubation, the substrate solution was added for colour development. The reaction was stopped by the addition of 2N sulfuric acid, and optical density was read at 450 nm in microplate reader. Each assay was performed in duplicate. Using the 4-parameter fit logistic (4-PL) curve equation, the actual concentration for each sample was calculated. The concentration for each sample was calculated after multiplying with the dilution factors to get the actual reading for each sample.

### 2.4. Statistical Analysis

Because of the large variances that we had in our data, we used the nonparametric Mann-Whitney test to compare means from two independent groups, and the nonparametric Kruskal-Wallis test was used for conducting analysis of variance (ANOVA) to compare means from more than two independent groups. Correlation between continuous variables was investigated by computation of the Pearson correlation coefficient. A *P* value less than 0.05 indicated statistical significance. Post-ANOVA pairwise comparisons of means were conducted using the Kruskal-Wallis test. For three groups, the critical *Z*-value for determining statistical significance was *Z* = 2.39. Exploratory logistic regression analysis, involving forcing entry into a logistic model the variables of interest, was conducted to identify the angiogenic and fibrogenic factors that had a significant association with the studied diseases. The mean level of each variable was used as the cut-off value for high versus low levels. SPSS version 15 and programs LR and 3S from Bio-Medical Data Processing Version 2007 (BMDP 2007) Statistical Software (Cork Technology Pack, Model Farm Road, Cord, Ireland) were used for the statistical analyses.

## 3. Results

### 3.1. Levels of Angiogenesis and Fibrogenesis Regulatory Factors in Vitreous Samples

 OPN, HMGB1, and PEDF were detected in all vitreous samples from patients with RD, PVR, and PDR. CTGF was detected in all vitreous samples from patients with PDR and PVR and in 11 (36.6%) samples from RD patients.

 Mean levels of OPN, HMGB1, CTGF, and PEDF in vitreous samples from PDR patients were significantly higher than those in RD patients (*P* < 0.001; *P* = 0.002; *P* < 0.001; *P* < 0.001, resp.; Mann-Whitney test). Mean levels of CTGF and PEDF in vitreous samples from PVR patients were significantly higher than those in RD patients (*P* < 0.001; *P* = 0.004, resp.; Mann-Whitney test). Mean levels of OPN and HMGB1 from PVR patients were higher than those in RD patients, but the differences between the means were not statistically significant (*P* = 0.425; *P* = 0.571, resp.; Mann-Whitney test) (Table 1).

### 3.2. Relationship between Angiogenesis and Fibrogenesis Regulatory Factors and Activity of PDR

 Comparison of mean levels of angiogenesis and fibrogenesis regulatory factors among active PDR patients, inactive PDR patients, and RD patients was conducted using the Kruskal-Wallis test, and the results are shown in Table 2. Mean levels differed significantly between the 3 groups for OPN (*P* < 0.001), HMGB1 (*P* = 0.002), CTGF (*P* < 0.001), and PEDF (*P* < 0.001). Post-ANOVA pairwise comparisons of means indicated that the mean OPN level was significantly higher in patients with active PDR than in RD patients (*Z* = 4.11). For HMGB1, the mean levels were significantly higher in patients with active PDR and patients with inactive PDR than in RD patients (*Z* = 2.92; *Z* = 2.97, resp.). For CTGF, the mean levels were significantly higher in patients with active PDR and patients with inactive PDR than those in RD patients (*Z* = 6.24; *Z* = 4.2, resp.). For PEDF, the mean levels were significantly higher in patients with active PDR and patients with inactive PDR than in RD patients (*Z* = 6.89; *Z* = 3.59, resp.). In addition, mean PEDF level was significantly higher in patients with active PDR than in patients with inactive PDR (*Z* = 2.57).

### 3.3. Relationship between Angiogenesis and Fibrogenesis Regulatory Factors and Traction Retinal Detachment

 When patients with PDR were divided into those with or without traction retinal detachment, the mean levels of angiogenesis and fibrogenesis regulatory factors differed significantly between PDR patients with traction retinal detachment, PDR patients without traction retinal detachment, and RD patients for OPN (*P* = 0.002), HMGB1 (*P* = 0.003), CTGF (*P* < 0.001), and PEDF (*P* < 0.001) (Table 3). Post-ANOVA pairwise comparisons of means indicated that, for OPN, the mean levels in PDR patients with or without traction retinal detachment were significantly higher than those in RD patients (*Z* = 5.18; *Z* = 5.64, resp.). For HMGB1, the mean levels in PDR patients with or without traction retinal detachment were significantly higher than those for RD patients (*Z* = 2.53; *Z* = 3.26, resp.). For CTGF, the mean levels in PDR patients with or without traction retinal detachment were significantly higher than those in RD patients (*Z* = 4.72; *Z* = 5.87, resp.). For PEDF, the mean levels in PDR patients with or without traction retinal detachment were significantly higher than those in RD patients (*Z* = 5.17; *Z* = 5.62, resp.).

### 3.4. Exploratory Logistic Regression Analysis

 PDR was significantly associated with high levels of HMGB1 (odds ratio = 7.39; 95% confidence interval = 2.11–25.9), CTGF (odds ratio = 11.4; 95% confidence interval = 2.87–45.3), and PEDF (odds ratio = 7.70; 95% confidence interval = 1.77–33.5). Active PDR was significantly associated with high levels of CTGF (odds ratio = 8.29; 95% confidence interval = 2.44–28.1) and PEDF (odds ratio = 7.66; 95% confidence interval = 2.00–29.4). PDR with traction retinal detachment was significantly associated with high levels of HMGB1 (odds ratio = 5.07; 95% confidence interval = 1.36–18.9).

 None of the studied angiogenesis and fibrogenesis regulatory factors was significantly associated with PVR.

### 3.5. Correlations

 In patients with PDR, there were significant correlations between the vitreous fluid levels of PEDF and the levels of OPN (*r* = 0.544, *P* = 0.0011), HGMB1 (*r* = 0.719, *P* < 0.001), and CTGF (*r* = 0.715, *P* < 0.001). In addition, there were significant correlations between the vitreous fluid levels of CTGF and the levels of OPN (*r* = 0.490, *P* = 0.002) and HMGB1 (*r* = 0.369, *P* = 0.027) (Table 4).

 In patients with PVR, there were significant correlations between the vitreous fluid levels of OPN and the levels of HGMB1 (*r* = 0.484, *P* = 0.049) and PEDF (*r* = 0.559, *P* = 0.020) (Table 4).

## 4. Discussion

 In this study, we examined the levels of the angiogenic and fibrogenic factors OPN, HMGB1, and CTGF and the antiangiogenic and antifibrogenic PEDF in the vitreous fluid from patients with PDR, PVR, and RD and their relationship with PDR clinical disease activity. We found upregulation of OPN, HMGB1, CTGF, and PEDF in the vitreous from PDR patients with active neovascularization compared with patients with quiescent PDR, PVR, and RD. Exploratory logistic regression analysis identified a significant association between PDR and high levels of HMGB1, CTGF, and PEDF, between active PDR and high levels of CTGF and PEDF, and between PDR with traction retinal detachment and high levels of HMGB1. Furthermore, there were significant correlations between the levels of PEDF and the levels of OPN, HMGB1, and CTGF in patients with PDR and between the levels of OPN and the levels of HMGB1 and PEDF in patients with PVR. 

In the present study, we report that OPN was significantly upregulated in the vitreous fluid from patients with PDR and that OPN levels were nonsignificantly elevated in the vitreous fluid from patients with PVR. In a previous study, Kase et al. [41] demonstrated increased levels of OPN in the vitreous fluid from patients with diabetic retinopathy; however, they studied only 11 cases. Our subgroup analysis showed that OPN levels in vitreous samples from active PDR cases were higher than those in quiescent cases. These results are in agreement with a previous report in which we demonstrated that OPN was expressed by vascular endothelial cells and stromal cells in PDR fibrovascular epiretinal membranes and by *α*-SMA-expressing myofibroblasts in PVR epiretinal membranes and that there was a significant correlation between the level of vascularization in PDR epiretinal membranes and the expression of OPN [42]. Taken together, these findings suggest a role for OPN in the progression of PDR.* In vitro *and *in vivo* studies demonstrated that OPN is an important angiogenic factor [15–18]. In addition, OPN is required for the activation, migration, proliferation, and differentiation of fibroblasts into *α*-SMA-expressing myofibroblasts [11–13] and is upregulated in several fibrotic diseases [9, 11, 12, 14]. Our results are consistent with previous reports showing that the proinflammatory cytokine OPN plays a role in the development of diabetic vascular complications [9, 43–45]. 

 The proinflammatory cytokine HMGB1 [20–23] exhibits angiogenic [24–27] and fibrogenic [28–31] effects. Another interesting role of HMGB1 in neovascularization is its ability to attract endothelial progenitor cells to sites of tissue injury and tumors to improve neovascularization [26]. Several studies showed overexpression of HMGB1 in other fibrotic disorders [28, 29, 31]. *In vitro* studies demonstrated that HMGB1 stimulated the proliferation and migration of fibroblasts [28, 30]. In addition, exposure of epithelial cells to HMGB1 resulted in the transition from an epithelial to myofibroblast-like phenotype, with a significant increase in the mesenchymal markers *α*-SMA and vimentin [31]. Recently, Arimura et al. [46] demonstrated that HMGB1 stimulated the migration of human retinal pigment epithelial cells. In the present study, we report that HMGB1 was significantly upregulated in the vitreous fluid from patients with PDR, particularly in patients with active neovascularization in agreement with our previous report [47]. Furthermore, exploratory logistic regression analysis demonstrated significant associations between high levels of HMGB1 and all PDR and PDR with traction retinal detachment. These findings suggest a role for HMGB1 in the progression of PDR. In addition, in this study, we demonstrated that PVR eyes had a 3-fold increase in the vitreous level of HMGB1 when compared with those with RD. These results are in agreement with a previous report in which we demonstrated that HMGB1 was expressed by *α*-SMA-positive myofibroblasts in PVR epiretinal membranes [42]. In addition to its role in mediating inflammation, angiogenesis, and fibrogenesis, several studies demonstrated that extracellular HMGB1 can aggravate tissue damage in neuronal tissue after ischemia [48–50].

 Recently, various studies suggested an important role for the proangiogenic [35, 36] and profibrotic [32–34, 51] CTGF in the development of chronic diabetes-related end-organ complications, including diabetic nephropathy [52]. In the present study, CTGF levels in the vitreous fluid from patients with PDR and PVR were significantly higher than those in the vitreous fluid from patients with RD. Our observations are consistent with previous reports showing increased CTGF levels in the vitreous fluid from patients with PDR [53–55] and PVR [54]. However, levels of CTGF in the vitreous fluid from patients with PDR were 3-fold higher than those in patients with PVR and levels of CTGF were particularly high in PDR patients with active neovascularization. In addition, exploratory logistic regression analysis demonstrated significant associations between high levels of CTGF and all PDR and PDR with active neovascularization. Our results are not in agreement with a previous report, in which Kuiper et al. [55] showed that CTGF levels in the vitreous fluid from patients with PDR were significantly associated with the degree of fibrosis. Our results are in agreement with a previous report in which we demonstrated increased expression of CTGF in the retinas from subjects with diabetes and that CTGF was expressed by vascular endothelial cells and *α*-SMA-expressing myofibroblasts in PDR epiretinal membranes and by myofibroblasts in PVR epiretinal membranes. In addition, there was a significant correlation between the level of vascularization in PDR epiretinal membranes and the expression of CTGF [6].

 PEDF has been shown to be the most potent endogenous inhibitor of angiogenesis. The activity of PEDF is selective in that it targets only new vessel growth and spares the preexisting vasculature [37, 38, 56]. The results of different studies on PEDF expression in the vitreous from patients with PDR are conflicting. Several studies found significant decrease in vitreous PEDF levels in patients with PDR [57–59]. Other studies found that PEDF levels in patients with PDR were not different from those in the controls [60, 61]. However, Duh and associates [62] demonstrated significant increase of PEDF levels in the vitreous from patients with active PDR. We do not have an explanation for the differences; however, differences maybe method related.

 In the present study, the levels of PEDF in the vitreous from patients with PDR and PVR were significantly greater than those in patients with RD. In addition, PEDF levels in the vitreous from patients with PDR were higher than those in patients with PVR. Our subgroup analysis showed that PEDF levels were significantly higher in the vitreous from patients with active PDR compared with patients with quiescent PDR. Furthermore, exploratory logistic regression analysis demonstrated significant associations between high levels of PEDF and all PDR and active PDR. Similarly, studies of other angiogenic eye diseases demonstrated increased levels of PEDF in the aqueous humor in patients with choroidal neovascularization [63] and macular edema secondary to branch retinal vein occlusion [64]. 

In the present study, we demonstrated that the vitreous fluids from patients with PDR and PVR express different regulators of angiogenesis and fibrogenesis. Recently, Lenga et al. [13] showed that OPN is required for the presence of HMGB1 in the focal adhesions of fibroblasts and for CTGF expression by fibroblasts in response to the proinflammatory cytokine transforming growth factor-*β*1. These findings suggest that HMGB1, and CTGF serve to mediate the immune response attributed to OPN and that the interaction between OPN, HMGB1, and CTGF modulates fibroblast functions. The significant positive correlations between PEDF levels and the levels of OPN, HMGB1, and CTGF in the vitreous from patients with PDR in the present study echoed the findings of Matsuoka et al. [65] that both PEDF and the angiogenic VEGF have been strongly expressed temporally and spatially in the retina of diabetic rats. Similarly, there was a significant positive correlation between the expression of VEGF and PEDF in patients with choroidal neovascularization [63]. Our findings suggest that increased levels of PEDF in the vitreous of patients with PVR and PDR, particularly active PDR, may be a response to counteract the activity of the angiogenic and fibrogenic factors. In addition, our data suggest that a positive regulatory feedback loop may exist in PDR, such that increased OPN, HMGB1, and CTGF induced synthesis of PEDF.

In conclusion, our data suggest that the upregulation of OPN, HMGB1 and CTGF contribute to the pathogenesis of proliferative vitreoretinal disorders and that increased levels of PEDF may counteract the activity of angiogenic and fibrogenic factors during the progression of PDR and PVR. The OPN/HMGB1/CTGF pathway maybe a novel therapeutic target to inhibit progression of PDR and PVR. 

## Figures and Tables

**Table 1:** Comparisons of mean angiogenesis and fibrogenesis regulatory factor levels in proliferative diabetic retinopathy (PDR), proliferative vitreoretinopathy (PVR), and rhegmatogenous retinal detachment (RD) patients.

Disease group	OPN (ng/mL)	HMGB1 (ng/mL)	CTGF (ng/mL)	PEDF (ng/mL)
PDR (*n* = 48)	837.36 ± 1012.3	4.47 ± 10.1	1.91 ± 2.2	4.06 ± 7.9
RD (*n* = 30)	209.33 ± 192.5	0.98 ± 0.9	0.22 ± 0.3	0.32 ± 0.2
*P* value (Mann-Whitney test)	<0.001*	0.002*	<0.001*	<0.001*
PVR (*n* = 17)	737.95 ± 996.5	2.79 ± 5.4	0.65 ± 0.5	0.98 ± 0.9
RD (*n* = 30)	209.33 ± 192.5	0.98 ± 0.9	0.22 ± 0.3	0.32 ± 0.2
*P* value (Mann-Whitney test)	0.425	0.571	<0.001*	<0.004*

*Statistically significant at 5% level of significance.

OPN: osteopontin; HMGB1: high-mobility group box-1; CTGF: connective tissue growth factor; PEDF: pigment epithelium-derived factor.

**Table 2:** Comparisons of mean angiogenesis and fibrogenesis regulatory factor levels in proliferative diabetic retinopathy (PDR) patients with or without active neovascularization.

Disease group	OPN (ng/mL)	HMGB1 (ng/mL)	CTGF (ng/mL)	PEDF (ng/mL)
Active PDR (*n* = 28)	882.54 ± 1024.4	5.48 ± 11.7	2.15 ± 2.4	4.98 ± 9.2
Inactive PDR (*n* = 20)	579.66 ± 816.0	2.74 ± 2.0	1.05 ± 1.1	1.69 ± 2.0
RD (*n* = 30)	209.33 ± 192.5	0.98 ± 0.9	0.22 ± 0.3	0.32 ± 0.2
ANOVA *P* value	<0.001*	0.002	<0.001*	<0.001*

*Statistically significant at 5% level of significance.

OPN: osteopontin; HMGB1: high-mobility group box-1; CTGF: connective tissue growth factor; PEDF: pigment epithelium-derived factor.

RD: rhegmatogenous retinal detachment.

**Table 3:** Comparisons of mean angiogenesis and fibrogenesis regulatory factor levels in proliferative diabetic retinopathy (PDR) patients with or without traction retinal detachment (TRD).

Disease group	OPN (ng/mL)	HMGB1 (ng/mL)	CTGF (ng/mL)	PEDF (ng/mL)
PDR with TRD (*n* = 21)	584.15 ± 910.9	4.08 ± 6.9	1.38 ± 1.9	3.18 ± 5.5
PDR without TRD (*n* = 27)	868.2 ± 962.6	4.57 ± 10.7	1.94 ± 2.1	3.94 ± 8.4
RD (*n* = 30)	209.33 ± 192.5	0.98 ± 0.9	0.22 ± 0.3	0.32 ± 0.2
ANOVA *P* value	0.002*	0.003*	<0.001*	<0.001*

*Statistically significant at 5% level of significance.

OPN: osteopontin; HMGB1: high-mobility group box-1; CTGF: connective tissue growth factor; PEDF: pigment epithelium-derived factor; RD: rhegmatogenous retinal detachment.

**Table 4:** Pearson correlation coefficients between variables in proliferative diabetic retinopathy (PDR) and proliferative vitreoretinopathy (PVR) patients.

Disease	Variable	PEDF	OPN	HMG1
PDR	OPN			
		*r* = 0.544		
		*P* = 0.001*		
	HMGB1			
		*r* = 0.719	0.255	
		*P* = <0.001*	0.145	
	CTGF			
		*r* = 0.715	0.490	0.369
		*P* = <0.001*	0.002*	0.027*

PVR	OPN			
		*r* = 0.559		
		*P* = 0.020*		
	HMGB1			
		*r* = 0.374	0.484	
		*P* = 0.140	0.049*	
	CTGF			
		*r* = 0.015	0.293	−0.033
		*P* = 0.953	0.253	0.899

*Statistically significant at 5% level of significance.

OPN: osteopontin; HMGB1: high-mobility group box-1; CTGF: connective tissue growth factor; PEDF: pigment epithelium-derived factor.
